# Surface characterization of the thermal remodeling helical plant virus

**DOI:** 10.1371/journal.pone.0216905

**Published:** 2019-05-31

**Authors:** Alexander L. Ksenofontov, Natalia V. Fedorova, Gennady A. Badun, Marina V. Serebryakova, Nikolai A. Nikitin, Ekaterina A. Evtushenko, Maria G. Chernysheva, Elena N. Bogacheva, Eugeny N. Dobrov, Ludmila A. Baratova, Joseph G. Atabekov, Olga V. Karpova

**Affiliations:** 1 Belozersky Institute of Physico-Chemical Biology, Lomonosov Moscow State University, Moscow, Russia; 2 Department of Chemistry, Lomonosov Moscow State University, Moscow, Russia; 3 Department of Virology, Lomonosov Moscow State University, Moscow, Russia; 4 Semenov Institute of Chemical Physics, Russian Academy of Sciences, Moscow, Russia; University of South Carolina, UNITED STATES

## Abstract

Previously, we have reported that spherical particles (SPs) are formed by the thermal remodeling of rigid helical virions of native tobacco mosaic virus (TMV) at 94°C. SPs have remarkable features: stability, unique adsorption properties and immunostimulation potential. Here we performed a comparative study of the amino acid composition of the SPs and virions surface to characterize their properties and take an important step to understanding the structure of SPs. The results of tritium planigraphy showed that thermal transformation of TMV leads to a significant increase in tritium label incorporation into the following sites of SPs protein: 41–71 а.a. and 93–122 a.a. At the same time, there was a decrease in tritium label incorporation into the N- and C- terminal region (1–15 a.a., 142–158 a.a). The use of complementary physico-chemical methods allowed us to carry out a detailed structural analysis of the surface and to determine the most likely surface areas of SPs. The obtained data make it possible to consider viral protein thermal rearrangements, and to open new opportunities for biologically active complex design using information about SPs surface amino acid composition and methods of non-specific adsorption and bioconjugation.

## Introduction

The phenomenon of tobacco mosaic virus (TMV) thermal transition at 94°C into RNA-free protein spherical particles (SPs) was revealed in our previous works [1]. We demonstrated that SPs are highly stable to various physical factors, water-insoluble and biodegradable. The SPs size (53 nm and over) depends on initial concentration of TMV and can be controlled. The SPs are safe for mammals, have unique adsorption properties and are an effective adjuvant [2–6]. Thus, SPs are able to serve as a basis for developing new pharmaceuticals, including vaccines [7–10]. However, the fine structure of the SPs has not yet been determined. Therefore, any additional information about the structure of the SPs can be important, as it will help to understand the origin of the unique properties of these particles and the possibilities of their application in biotechnology.

To characterize the surface area of proteins of intact virions and SPs, we subjected the SPs preparations to atomic tritium labelling. The TMV coat protein (CP) surface, accessible for tritium labelling in native virions (U1) has been described previously [11, 12].

This method has been used successfully to determine the surfaces of proteins in different macromolecular complexes, including plant and animal viruses, ribosomes, cells and membrane proteins [11, 13–19].

Technique based on nonselective replacement of protium for tritium in a superficial layer of a macromolecule (on depth 3–5 Å). The introduction of tritium label in biological compounds is realized by the bombardment with the beam of hot tritium atoms of the target cooled down by fast freezing of protein solution and virus particles at liquid nitrogen temperature. The vessel-reactor was evacuated, and the generation of hot tritium atoms was attained by the catalytic dissociation of molecular tritium at the tungsten wire heated up to 2000 K. The introduction of tritium labels under such conditions occurs through single collisions of tritium atoms with the target, and the intramolecular distribution of the labels among the residues of amino acids is determined by their accessibility in a macromolecule [20].

Earlier, we compared the distribution of the tritium label in wild type TMV virion (strain U1) and temperature-sensitive TMV mutant ts21-66 [12] and found the screening of the N-terminal CP segment. The purpose of present work was a comparative study of the amino acid composition of the SPs and virions surface to characterize their properties. In this study, the SPs segments accessible to tritium labeling were identified. A comparison of the locations of tritium label in protein amino acids in the intact TMV virion and SPs was made in order to determine structural fragments of CP involved in transformation and the most likely surface areas of SPs. We used independent experimental approaches, namely limited proteolysis and MALDI-TOF mass spectrometer (MS) analysis of the proteolysis products of SPs and virions, to confirm our results.

## Materials and methods

### Virus and SPs preparation

The detailed protocol for extraction and purification of TMV from the infected *Nicotiana tabacum L*. *cv*. *Samsun* leaves was described previously [21]. SPs samples were prepared from TMV according to [1]. The morphology and size distribution of the obtained TMV spherical particles were subsequently analyzed by transmission electron microscopy and nanoparticle tracking analysis according to [22, 23]. The average size of SPs was 260 nm.

### Fluorescence labelling

The procedure of fluorescein isothiocyanate (FITC) labelling of TMV virions and SPs was carried out according to the manufacturer’s protocol (Sigma), with some modifications [2]. For FITC-labelling experiments in addition to the SPs with an average size of 260 nm, 980 nm SPs were used. The results of labelling were detected by fluorescence microscopy using an Axiovert 200M fluorescence microscope (Carl Zeiss, Germany) equipped with a digital cooled camera ORCAII-ERG2 (Hamamatsu, Japan) and SDS-PAGE electrophoresis, with subsequent analysis in UV light with a ChemiDOC XRS+ (Bio-Rad Laboratories, USA) accordingly [2].

### Tritium bombardment

The labelling of intact viruses and SPs using hot tritium atoms was carried out in the same way as described earlier [11, 13]. Tritium was introduced into the surface layer of the samples by bombardment of hot tritium atoms at the temperature up to 2000 K. A thin film of the suspension (0.5 mL at 1 mg • mL^– 1^) in 5 mM phosphate buffer, pH 7.0, was formed and lyophilized by the inside of a cylindrical glass reactor cooled to liquid nitrogen temperature. Three 10-second molecular tritium (^3^H_2_) pumping up when filling the reaction vessel to a pressure of 0.5 Pa were used to bombard samples with atomic tritium. The virus and SPs samples were washed with 5 mM phosphate buffer, pH 7.0 after labeling, and the SPs samples were lyophilized twice to clean samples from labile tritium. Both viruses and SPs retained their integrity, judging by the data of transmission electron microscopy and PAGE.

### Analytical methods

Trypsinolysis was carried out at the enzyme: substrate ratio of 1:100 (w/w) for 1 h at 37°C in 5 mM Tris pH 7.8. Then the hydrolysis was continued for another 4 hours with a new portion (1:50 w/w) of trypsin (Promega). Samples, dissolved in 0.1% trifluoroacetic acid (TFA), were separated on an Ultrasphere ODS column (5 μm), 250 × 4.6 mm i.d. (Beckman) in a Beckman 344 chromatograph at flow rate of 1 mL·min^−1^, with gradients of acetonitrile (in 60 min 0–50% and in 10 min 50–60%), with 0.1% TFA. Eluate fractions were collected from 8–10 separations. The peptides were acid hydrolyzed as described previously [24]. Amino acid analysis with cation-exchange column and ninhydrin derivatization with our modifications [25] was performed on a Hitachi L8800 analyzer. We used a 150TR Radiomatic flow scintillation analyzer (Packard Co.) for simultaneous analysis of tritium radioactivity of amino acid. Data were processed using MultiChrom 1.71a (Ampersand Ltd., Moscow, Russia). The molar radioactivity for each amino acid residue in the peptide was calculated [26].

### Limited proteolysis of SPs and virions and MALDI TOF-TOF MS analysis

Limited proteolysis of the native virions and SPs was performed at 25°C using trypsin (Promega). All samples were treated with trypsin in enzyme/substrate ratios of 1:500 (wt/wt), for different time, as described by us previously [27] and subjected to MS analysis. MALDI TOF-TOF Ultraflex II mass spectrometer (Bruker Daltonik, Germany) with a 355 nm laser (Nd) was used to determine the mass spectra. The software GPMAW 4.04 and MASCOT Peptide Mass analysis (www.matrixscience.com) were applied to search for correspondences between the observed ratio of mass to charge (m/z) and peptides.

## Results

While the structure of TMV virions is available at 3.6 Å resolution [28], which allows the identification of solvent-exposed surface amino acids, this information is not available at the present time for SPs. Our previous work has shown considerable conformational changes occur within the coat protein during the TMV to SPs thermal transition [29]. The transition into SPs is accompanied by a partial loss of α-helical protein structure and emergence of a β-sheet structure, giving the particles a high density. Thus, there are significant structural differences comparing TMV and SPs.

### FITC-labelling of SPs and TMV virions

**Fig 1:**
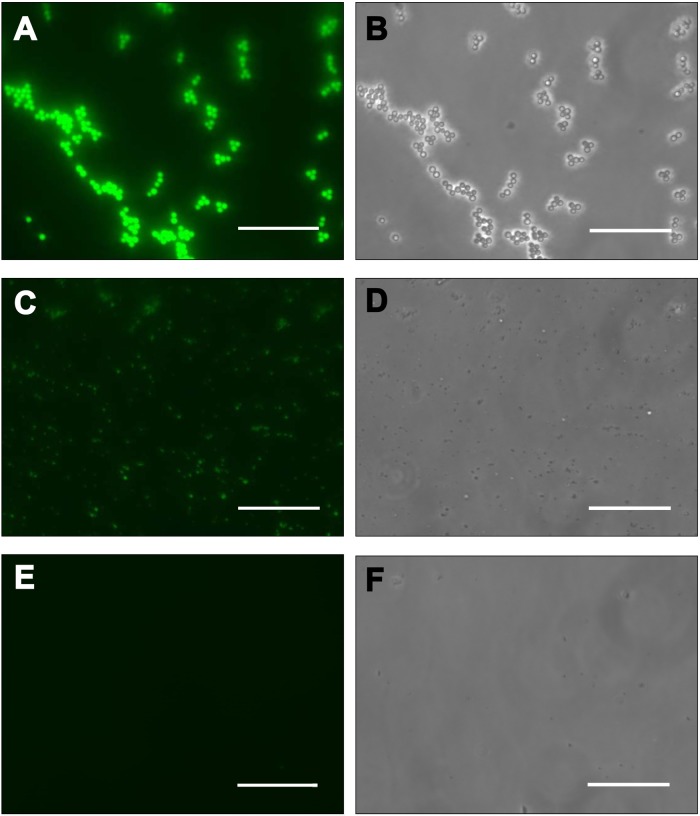
Fluorescein isothiocyanate (FITC) binds to the surface of SPs. (A, B) SPs (980 nm in diameter); (C, D) SPs (260 nm in diameter); (E, F) native TMV virions—control. (A, C, E) fluorescence microscopy, (B, D, F) phase contrast. Bars, 3 μμm.

Firstly, we used fluorescein isothiocyanate to label the surface of native virions and SPs. Tobacco mosaic virus have no chemical reactive amino acids on the virions surface and have post-translation modification of CP N-terminus (N-acetyl serine) [30, 31]. In this regard, we were not surprised that TMV is not labeled with FITC (Fig 1E, S1 Fig line 6). However, after FITC-labeling of SPs we detected fluorescence of particles (Fig 1A and 1C) and SPs protein in UV-light (S1 Fig lines 4–5). Presumably, under our reactions conditions (0.1 M sodium carbonate buffer, pH 9.1) lysine amino acid residues (K53 and K68) are labeled. We suppose that thermal denaturation of TMV leads to appearance on SPs surface of one or two lysines capable of reacting with FITC. These data suggest a difference in the amino acid composition of the SPs and native virions surface.

### Tritium planigraphy study of spherical particles

A comparative study by tritium planigraphy of two kinds of preparation was carried out: the intact TMV virions and SPs. In preliminary experiments, we investigated different conditions of labelling of SPs preparations (types of targets, reaction temperature), to increase the effectiveness of their labelling. It had been shown that the freezing-lyophilization operation does not lead to any changes in the spherical particles. SPs retained their integrity, as judged by transmission electron microscopy and PAGE. The distribution of tritium in amino acids of protein SPs labelled under different conditions after total acid hydrolysis was maintained, which also confirmed the preservation of the structure of SPs.

**Fig 2:**
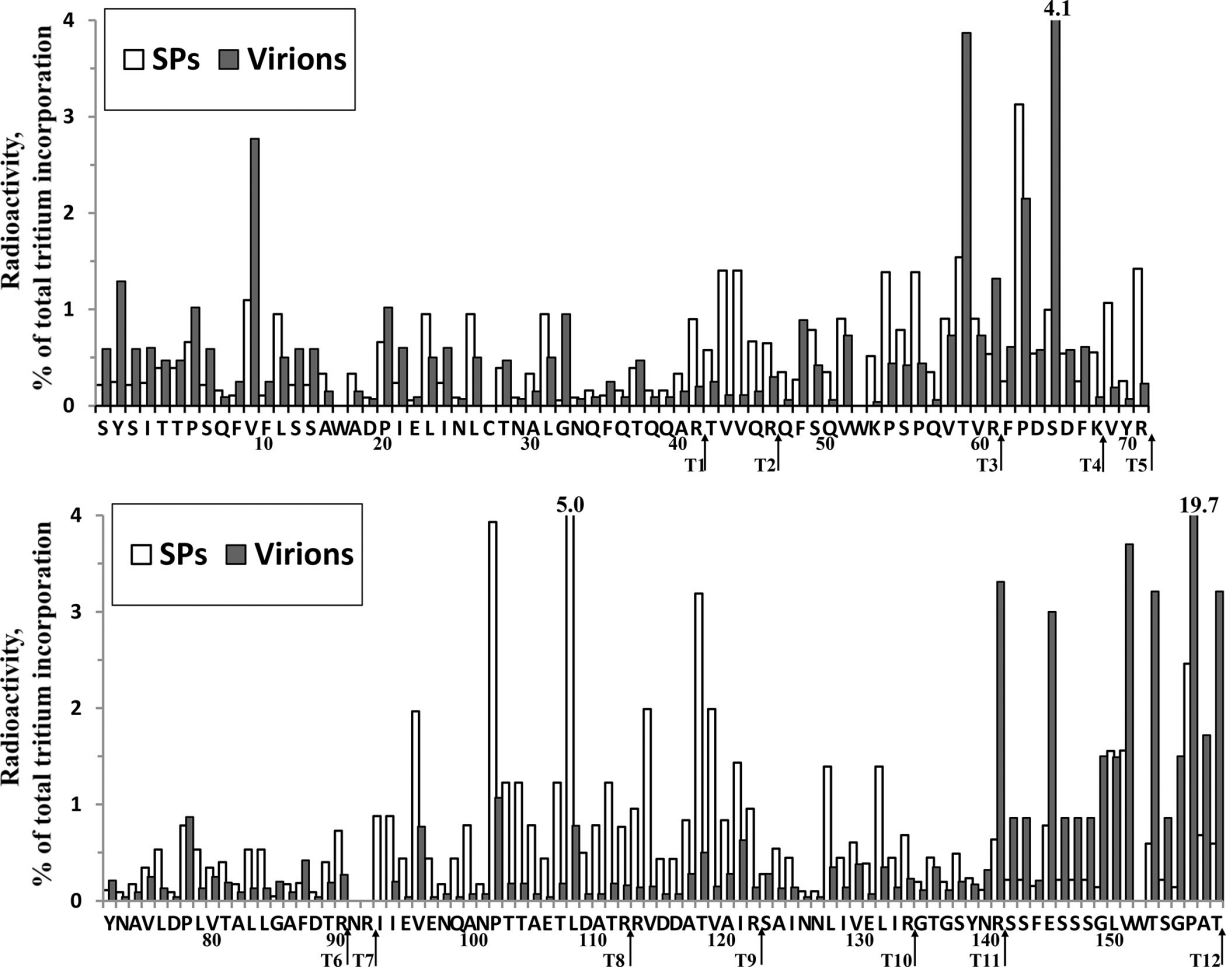
The tritium label location in the TMV virion and SPs proteins upon tritium bombardment of samples. A_i_/A_Σ_ (%), the ratio of the specific radioactivity of the residue (A_i_) to the overall radioactivity of all protein residues (A_Σ_), in %. Amino acid residues numbering (in a single-letter code) is shown at the bottom of the axis. The trypsin cleavage sites are shown by arrows. The data for cysteine and tryptophan are not shown because they were destroyed by acid hydrolysis.

The location of tritium label along the polypeptide chain of TMV and SPs after tritium labelling of the intact SPs and virions preparation is shown in Fig 2. Data for the TMV virus are those published in our previous work [12]. Two repeated experiments on the tritium bombardment of the SPs were carried out, with resembling results. The location of label in virions and SPs differ significantly.

The label distribution between the tryptic peptides is given in Table 1. Surface regions of SPs and virions are seen more clearly in columns 6 and 7, respectively, where relative radioactivity has been calculated per amino acid in peptide. The tritium label in the intact virus was mainly included in the surface sites of the polypeptide chain, according to X-ray diffraction [28]—the N-terminal region (18%), the C-terminal region (≈45%) and the Thr59–Ser65 region (≈10% of total label) [12]. A five-fold increase in label inclusion in the SPs in comparison with virions occurred in T2 (Thr42–Arg46), T5 (Val69–Arg71) and T8-T10 (Ile93– Arg134) peptides, and a pronounced drop (four-fold) occurred in T11-12 (Gly135–Thr158) peptides. The labeling of protein areas that form axial or lateral intersubunit contacts in the virion (located in peptides T2, T5, T6, T8, T9 and T10) has greatly decreased in SPs, while the labeling of areas exposed on the surface of the virion (located in peptides T1, T3, T4, T11 and T12) increased. A similar consistent pattern was observed earlier [12] when comparing labeling of the virion and isolated protein. The Table 1 also shows the tritium distribution between the tryptic peptides of the CP after labelling of isolated CP preparations, published in our work [12]. A two-fold increase in label inclusion in the SPs in comparison with CP`s occurred in T2-T4 (Thr42– Lys68) and a two-fold drop occurred in T10 (Ser123–Arg134), T12 (Ser142–Thr158) peptides. Thus, our data indicate a significant difference in amino acids surface composition of SPs, virions and CP isolated from TMV.

**Table 1:** Tritium label distribution between tryptic peptides of SPs, TMV virion and isolated CP.

Peptide (amino acids)	Number of residues	Total peptide radioactivity, %			Averaged peptide radioactivity^a^, %/ residue			Relative radioactivity change^b^,	
		SPs	Virions	CP	SPs	Virions	CP	A_SPs_/A_virion_	A_SPs_/A_CP_
T1 (Ser1–Arg41)	39	13,6	18,0	17,0	0,3	0,5	0,4	0,8 (0,1)	0,8 (0,1)
T2 (Thr42–Arg46)	5	4,7	0,9	2,7	0,9	0,2	0,5	5,2 (2,0)	1,7 (0,7)
T3 (Glu47–Arg61)	14	11,0	10,2	4,9	0,8	0,7	0,4	1,1 (0,2)	2,2 (0,5)
T4 (Phe62–Lys68)	7	6,3	8,7	2,7	0,9	1,2	0,4	0,7 (0,2)	2,3 (0,7)
T5 (Val69–Arg71)	3	2,7	0,5	3,1	0,9	0,2	1,0	5,5 (2,0)	0,9 (0,4)
T6 (Tyr72–Arg90)	19	6,3	3,7	4,6	0,3	0,2	0,2	1,7 (0,4)	1,4 (0,1)
T7 (Asn91–Arg92)	2	-	-	-	-	-	-	-	
T8 (Ile93–Arg112)	20	23,3	4,3	16,3	1,2	0,2	0,8	5,4 (0)	1,4 (0)
T9 (Arg113–Arg122)	10	13,1	2,4	12,9	1,3	0,2	1,3	5,4 (0,2)	1,0 (0)
T10 (Ser123–Arg134)	12	6,8	2,3	11,2	0,6	0,2	0,9	3,0 (0,4)	0,6 (0,1)
T11 (Gly135–Arg141)	7	2,3	4,6	2,2	0,3	0,7	0,3	0,5 (0,1)	1,1 (0,2)
T12 (Ser142–Thr158)	16	10,0	44,4	22,4	0,6	2,8	1,4	0,2 (0)	0,4 (0,1)
Sum	154	100	100	100					

^a^ Average peptide radioactivity was calculated by dividing the total radioactivity of the peptide by the number of residues constituting the peptide, i.e. an average radioactivity of one residue in a peptide was determined.

^b^ The ratios of the radioactivity of the SPs, virions and CP peptides are shown. Standard deviations are given in parentheses.

Possible surface regions of the SPs included two segments of about thirty amino acids: Arg41-Arg71, T2-T5 (≈26% of total label for SPs) and Ile93–Arg122, T8-T9 (≈36%).

Possibly, the four-helix α-helical bundle in the TMV CP subunit [28] partially unfolds in the process of TMV to SPs transition. Tritium label incorporation among protein secondary structure elements is calculated by us and presented in Table 2 for the virions, SPs and isolated CP preparations.

**Table 2:** Tritium label incorporation among protein secondary structure elements in the virion, SPs and isolated CP.

Secondary structure element	Amino acid residues	Number of residues	Summarized element radioactivity ^a^, %			Averaged element radioactivity ^b^, %/residue			Relative radioactivity change,			
			SPs	Virions	СP	SPs	Virions	CP	A_SPs_/A_virion_		A_SP_/A_CP_	
*l*1	`1–8	8	2,6	5,6	2,4	0,32	**0,70**	0,31		0,5		1,1	
α1	`9–14	6	2,6	4,5	7,1	0,44	**0,74**	**1,18**		0,6		0,4	
*l* 2	15–16	2	0,5	0,7	0,3	0,27	0,37	0,14		0,7		2,0	
β1	17–18	2	0,3	0,2	0,3	0,17	0,08	0,13		2,2		1,3	
α2	19–33	15	5,2	5,7	6,4	0,34	0,38	0,42		0,9		0,8	
*l* 3	34–36	3	0,4	0,4	0,2	0,14	0,14	0,06		1,0		2,2	
α3	37–52	16	9,3	4,1	4,6	**0,58**	0,26	0,29		2,3		2,0	
*l* 4	53–67	15	14,0	16,7	6,2	**0,93**	**1,11**	0,41		0,8		2,3	
β2	68–70	3	1,9	0,4	2,4	**0,63**	0,12	**0,80**		5,4		0,8	
*l* 5	71–72	2	1,5	0,4	0,8	**0,77**	0,22	0,41		3,5		1,9	
α4	73–86	14	4,7	2,6	3,6	0,34	0,19	0,26		1,8		1,3	
*l* 6	87–110	24	22,7	4,9	16,3	**0,95**	0,20	**0,68**		4,7		1,4	
α5	111–135	25	22,1	5,2	24,9	**0,88**	0,21	**1,00**		4,3		0,9	
*l* 7	136–137	2	0,6	0,5	0,7	0,32	0,23	0,33		1,4		1,0	
β3	138–139	2	0,7	0,4	0,5	0,36	0,19	0,24		2,0		1,5	
α6	140–146	7	2,3	9,4	2,1	0,33	**1,35**	0,31		0,2		1,1	
C	147–158	12	8,4	38,6	21,3	**0,70**	**3,22**	**1,78**		0,2		0,4	

^a^ Summarized element radioactivity was evaluated by summing radioactivity of all amino acids comprising an element.

^b^ Average element radioactivity was calculated by dividing the summarized element radioactivity by the number of residues comprising an element, i.e. an average radioactivity of one residue in an element was evaluated, and values above 0.55% are underlined and highlighted in bold.

Indeed, as seen in the Table 2, the segment in which there is an increase in the label content consists of an α3-helix (37–52), probably disordered, and an entire loop l4-β2-l5 (53–72). In addition, the other long segment, including a loop (87–110) and a possible disordered α5–helix (111–135), was also transformed to the surface of SPs. On the other hand, the inclusion of the label in the N- and C-ends (1–15, 142–158) was significantly reduced.

### Limited proteolysis of SPs and intact virions

**Fig 3:**
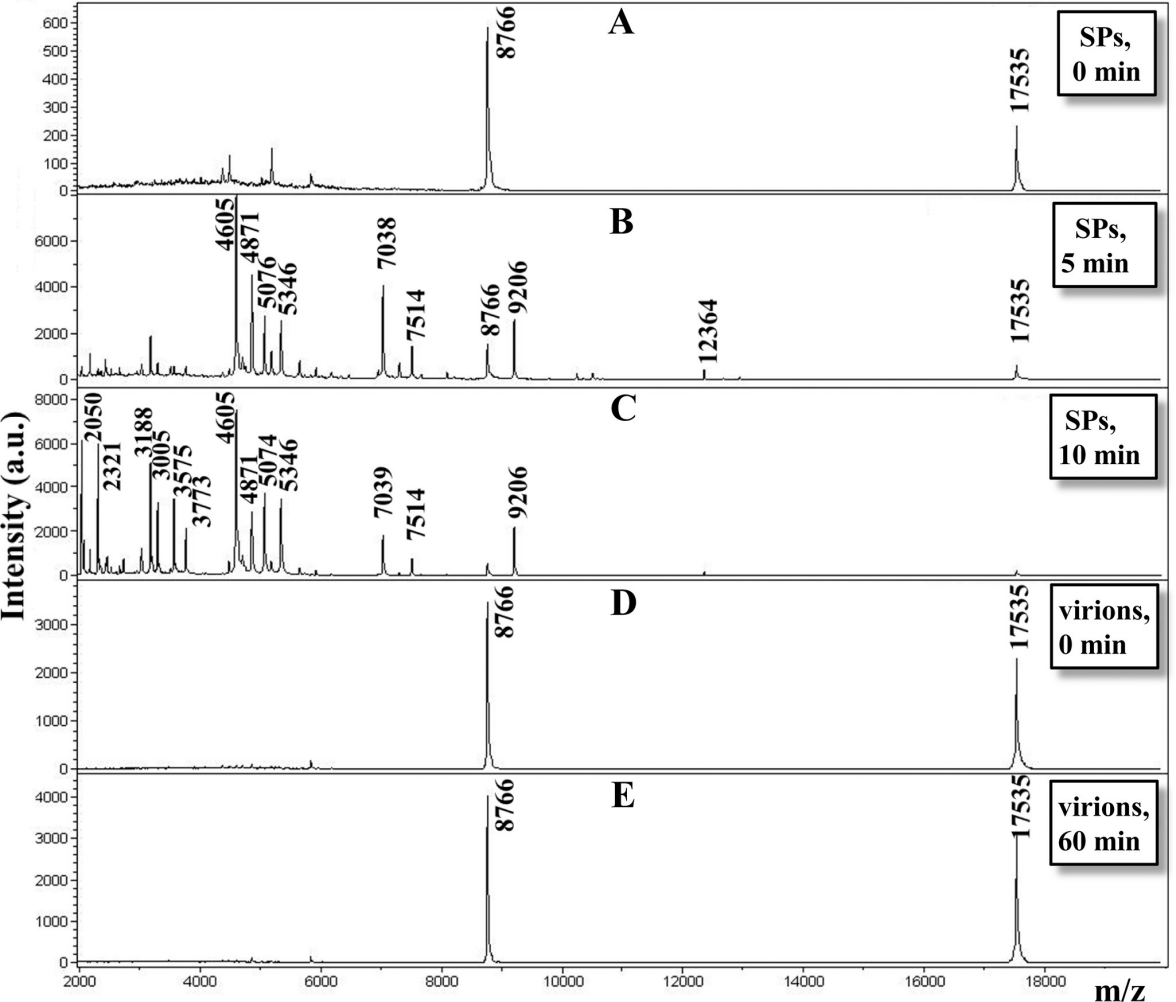
**MALDI-TOF MS analysis of the products of limited proteolysis of the proteins in SPs (A, B, C) and TMV virions (D, E).** Samples were incubated with trypsin in enzyme/substrate ratios of 1:500 for varying periods of time; incubation times at room temperature are shown. The molecular masses of the products of limited proteolysis are indicated above the peaks.

For a further characterization of SPs and comparison with virions of TMV, we used limited proteolysis by digestion with trypsin (in enzyme/substrate ratios 1:500, at room temperature, concentration of protein was 1 mg/mL). Fig 3 depicts the time course of proteolytic digestion of SPs (A, B, C) and intact virions (D, E), as monitored by MALDI-TOF MS analysis of the products. Intact virions did not digest within 1 hour of trypsinolysis. Proteolysis of SPs under the same conditions was observed on almost all tryptic sites, besides K68 and R134. Complete disappearance of an initial protein peak (17.5 kDa) occurred after 20 min of incubation. After 5 min, trypsinization peaks appeared with m/z 12364 [47–158], 7514 [47–112], 7038 [93–158], 5346 [47–92], 5076 [47–90], 4871 [113–158], 4605 [1–41] and 3189 [113–141] (Fig 3B). After 10 min of trypsinization, additional peaks appeared with m/z 3773 [123–158], 3575 [62–92], 3305 [62–90], 2321 [72–92] and 2050 [72–90] (Fig 3C).

These data suggest that limited trypsinolysis of the SPs induced step-by-step fragmentation of protein. The list of m⁄z values found and their correspondence with the database CP TMV protein sequence are summarized in Table 3.

**Table 3:** Peptides are products of limited proteolysis of the SPs with trypsin identified by MALDI-TOF MS analysis.

m/z	Amino acid residues	Intensity ^a^	
		5 min	10 min
17535	1–158	592	274
12364	47–158	389	125
9206	1-41-S-S-1-41	2583	2232
8768	1–158^b^	1501	581
7514	47–112	1411	800
7039	93–158	4090	1877
5346	47–92	2522	3500
5074	47–90	2750	3757
4869	113–158	4550	2915
4603	1–41	8476	7523
3773	123–158	-	2175
3575	62–92	-	3491
3305	62–90	-	3350
3188	113–141	1887	5111
2321	72–92	-	5952
2050	72–90	-	6154

^a^ -The intensity of the peaks from Fig 3B and 3C) and incubation time with trypsin are shown.

^b^—(MH++) ions of TMV protein.

The Table shows that trypsin, in 5 min, cleaved more than three protein sites of SPs (R41, R46, R112), and in 10 min of proteolysis cleavage, additional proteolysis sites appeared (R61, R71, R90, R92, R122, R141). We did not observe cleavage in K68 and R134, even after 1 hour of trypsinolysis.

These data, to some degree, correspond to our data obtained from a tritium planigraphy study, which are represented in Fig 2. The enzyme cleaved strongly labelled segments in the first 2–5 min, and weakly labelled segments in only 10 min. The obtained data indicate that these strongly labelled regions were located outside the globule, on the SPs surface and ⁄ or weakly structured, unlike the virion.

## Discussion

Transition of tobacco mosaic virus (TMV) into spherical particles (SPs) by thermal treatment at 94°C has been described in detail recently [1]. Previously, we studied some physicochemical properties of SPs using circular dichroism (CD), fluorescence spectroscopy and Raman spectroscopy [29]. However, the fine structure of the SPs had not yet been determined.

The results of fluorescence microscopy and SDS-PAGE electrophoresis of FITC-labelled TMV virions and SPs demonstrate that the surface amino acid composition of SPs and TMV is dramatically different. Here, a comparative tritium planigraphy study of the surface structure of SPs and virions, to characterize their surface and determine surface regions, was performed. It was established that the incorporation of tritium labels in SPs in some segments of protein differed from that of native virions. It has been shown that thermal transformation of TMV leads to a significant increase in tritium label inclusion into the 41–71 and 93–122 segments of the 158-residue-long protein, with some decrease in label introduction into the N- and C-terminal region (1–15, 142–158). The segments of the polypeptide chain available for tritium inclusion of TMV, SPs, isolated CP and schematic representation of the surface sites of SPs protein are indicated on S2 Fig. In the virion, the main label was close to the exterior part of the molecule [12, 28]. The increase in the availability of tritium in areas lying in the depth of the virion can be explained only by significant conformational changes of secondary and tertiary structures of the protein in SPs.

We also used independent experimental approaches—limited proteolysis and MALDI-TOF mass spectrometer analysis of the proteolysis products of SPs to explain the high level of tritium label incorporation into these areas. The cleavage sites were identified by a proteomic technique based on MALDI-TOF MS analysis. The enzyme cleaved strongly labelled segments in the first 2–5 min. Obtained data indicate that these strongly labelled regions were located on the SPs surface and ⁄ or weakly structured, unlike the virion. Previously, we found that unstructured segments in proteins were strongly labelled and easily cleaved by proteases [27, 32, 33]. In addition, using CD spectroscopy in the far UV range [29], we showed that SPs have a low α-helix content and a significant proportion of the β-structure, in contrast to intact virions (see S2 Fig). These results suggest that the TMV to SPs remodeling leads to changes in the amino acid composition exposed on the particles surface. Using fluorescence spectroscopy [29], we compared TMV and SPs spectra and found that the maximum position of the fluorescence spectrum shifted substantially (from 326 to 340 nm), indicating that after thermal remodeling the environment of Trp and Tyr residues altered to much more hydrophilic. Indeed, according to tritium planigraphy data (Fig 2), the aromatic residues Tyr 70, 72 and Trp 52 are included in the protein site, in which there is an increase in the inclusion of the label. Furthermore, reaction of SPs with thioflavin T suggests the appearance of cross-β-structures and amyloid-like structures. It is likely that, with the emergence of thermal associations of the TMV coat protein, the newly formed β-structures are involved in interactions between subunits, as we have shown for thermal denaturation of CP potato virus A [34] and potato virus X [35].

**Fig 4:**
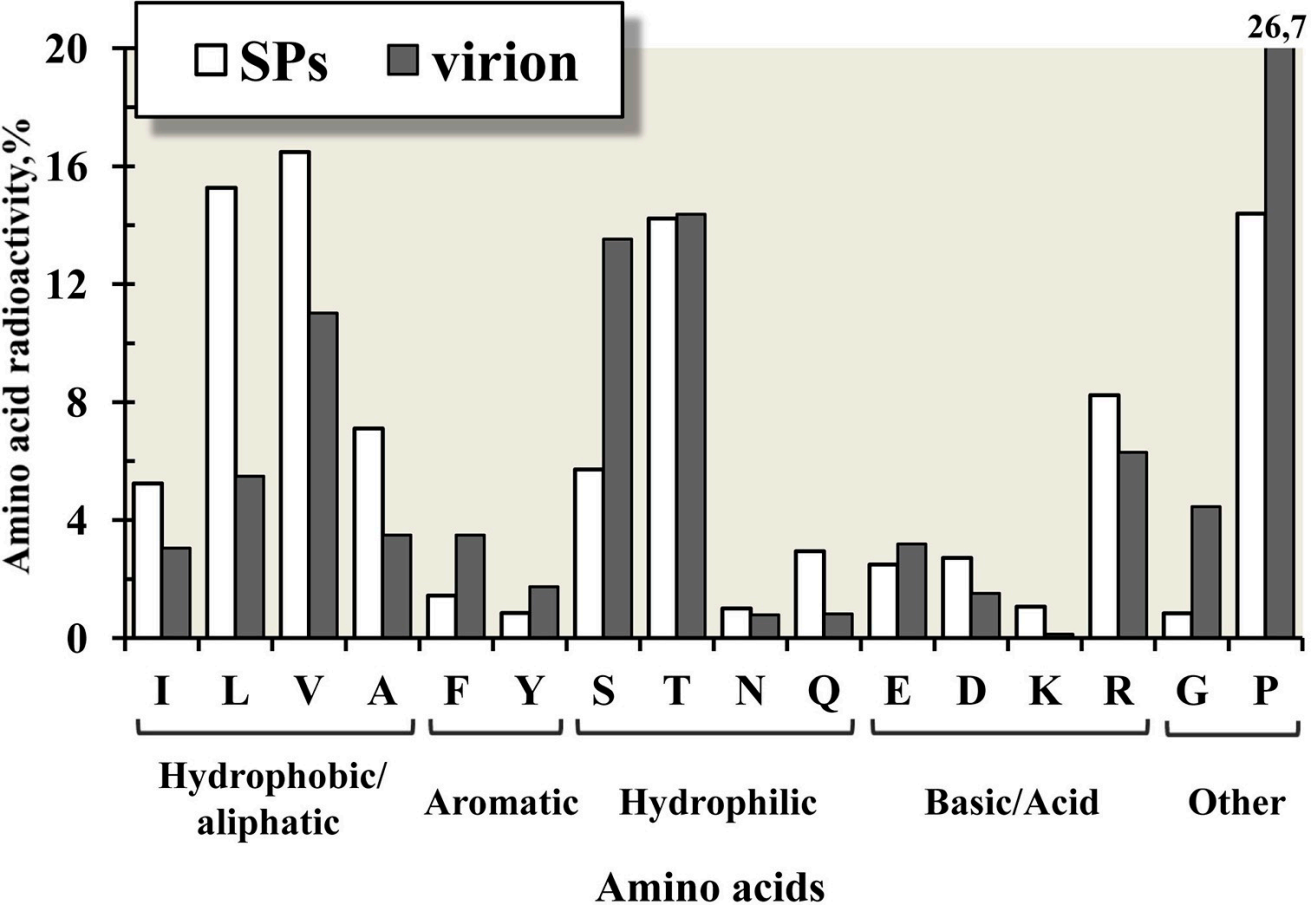
Total radioactivity of amino acid residues of protein in the SPs and virions of TMV. The data of residue radioactivity (in %) are presented for five groups: hydrophobic/aliphatic (I, L, V, A), aromatic (F, Y), hydrophilic (S, T, N, Q), basic/acid (E, D, K, R) and other (G, P) amino acids. The data on SPs TMV are shown as white columns, and the data on intact TMV as grey columns.

Apparently, assembly from thermally denatured protein subunits makes the SPs surface substantially hydrophobic. The total radioactivity of amino acid residues of protein in the SPs and virions of TMV is shown in Fig 4. The data of residue radioactivity (in %) are presented for five groups: hydrophobic/aliphatic (I, L, V, A), aromatic (F, Y), hydrophilic (S, T, N, Q), basic/acid (E, D, K, R) and other (G, P) amino acids. The figure shows a significant increase in the tritium labelling of hydrophobic/aliphatic residues I, L, V, A (by 1.5–3 times).

A recent study showed that SPs could be non-covalently modified through adsorption of foreign proteins on the SPs surface, and that these modifications are driven, supposedly, through electrostatic and hydrophobic interactions [2]. In addition, the total radioactivity of lysines increased by 8 times (Fig 4). Lysines 53 and 68 are presumably located in areas located on the SPs surface. These data are in agreement with the results of FITC-labelling and the data of the study, which investigated the reactivity of SPs to bioconjugation reactions directed to lysine [36].

In subsequent experiments, the secondary structure elements forming SPs will be analyzed, to build an accurate structure model of this protein.

The results of our work demonstrate the differences in the surface of the TMV SPs and virions and explain the unique properties of the TMV SPs. Data on changes in the amino acid composition of the surface enable a significant step to be taken towards understanding structural changes during the thermal transition of a virion-spherical particle, and open new possibilities for biologically active groups labelling on the SPs surface for novel bioengineering and medicine developments.

## Supporting information

S1 Fig SDS-PAGE of FITC-labeled SPs. (1, 4) SPs (980 nm in diameter); (2, 5) SPs (260 nm in diameter); (3, 6) native TMV virions—control. (1–3) Coomassie Blue staining, (4–6) UV light. Positions of the molecular weight markers (kDa) are indicated at the left side.
(TIF)

S2 Fig Schematic representation of the surface sites of SPs protein. **(**A) The tritium incorporation into amino acid residues along the polypeptide chain of proteins SPs, TMV and isolated CP. The coloring was carried out according to the principle of rainbow and reduction of specific activity (red> yellow> green> blue–matched >0.5%, 0.5–0.4%, 0.4–0.3%, 0.3–0%, respectively. The residues lost during analysis are not colored. α-Helices (H) and β-structure (B) in U1 CP are represented. (B) The surface sites of SPs protein are indicated. The figure is based on X-ray diffraction data TMV coat protein subunit in the virion [28]. Red color shows labeled area, that possibly transformed into betta and disordered structures [29]. Region with a low level of labeling is shown in gray.
(TIF)

S1 Dataset Underlying data used to reach the conclusions drawn in the manuscript. Data set for Fig 2 and Fig 4.
(PDF)

## References

[1] Atabekov J, Nikitin N, Arkhipenko M, Chirkov S, Karpova O. Thermal transition of native tobacco mosaic virus and RNA-free viral proteins into spherical nanoparticles. The Journal of general virology. 2011;92(Pt 2):453–6. 10.1099/vir.0.024356-0 . 20980527

[2] Karpova O, Nikitin N, Chirkov S, Trifonova E, Sheveleva A, Lazareva E, et al Immunogenic compositions assembled from tobacco mosaic virus-generated spherical particle platforms and foreign antigens. The Journal of general virology. 2012;93(Pt 2):400–7. 10.1099/vir.0.036293-0 . 22049093

[3] Nikitin NA, Zenin VA, Trifonova EA, Ryabchevskaya EM, Kondakova OA, Fedorov AN, et al Assessment of structurally modified plant virus as a novel adjuvant in toxicity studies. Regulatory toxicology and pharmacology:RTP. 2018;97:127–33. 10.1016/j.yrtph.2018.06.010 . 29932979

[4] Nikitin NA, Zenin VA, Trifonova EA, Ryabchevskaya EM, Yurkova MS, Kondakova OA, et al Data in support of toxicity studies of structurally modified plant virus to safety assessment. Data in brief. 2018;21:1504–7. 10.1016/j.dib.2018.10.102 30510980 PMC6258244

[5] Nikitin NA, Matveeva IN, Trifonova EA, Puhova NM, Samuylenko AY, Gryn SA, et al Spherical particles derived from TMV virions enhance the protective properties of the rabies vaccine. Data in brief. 2018;21:742–5. 10.1016/j.dib.2018.10.030 30406165 PMC6214837

[6] Nikitin NA, Trifonova EA, Karpova OV, Atabekov JG. Biosafety of plant viruses for human and animals. Moscow University Biological Sciences Bulletin. 2016;71(3):128–34. 10.3103/s0096392516030081

[7] Trifonova E, Nikitin N, Gmyl A, Lazareva E, Karpova O, Atabekov J. Complexes assembled from TMV-derived spherical particles and entire virions of heterogeneous nature. Journal of biomolecular structure & dynamics. 2014;32(8):1193–201. 10.1080/07391102.2013.816868 . 24099636

[8] Trifonova EA, Zenin VA, Nikitin NA, Yurkova MS, Ryabchevskaya EM, Putlyaev EV, et al Study of rubella candidate vaccine based on a structurally modified plant virus. Antiviral research. 2017;144:27–33. 10.1016/j.antiviral.2017.05.006 . 28511994

[9] Atabekov JG, Nikitin NA, Karpova OV. New type platforms for in vitro vaccine assembly. Moscow University Biological Sciences Bulletin. 2016;70(4):177–83. 10.3103/s0096392515040045

[10] Kondakova OA, Trifonova EA, Arkhipenko MV, Nikitin NA, Atabekov JG, Karpova OV. Development of Avian Influenza Vaccine on the Basis of Structurally Modified Plant Virus. Sel'skokhozyaistvennaya Biologiya. 2017;52(4):731–8. 10.15389/agrobiology.2017.4.731eng

[11] Goldanskii VI, Kashirin IA, Shishkov AV, Baratova LA, Grebenshchikov NI. The use of thermally activated tritium atoms for structural-biological investigations: the topography of the TMV protein-accessible surface of the virus. Journal of molecular biology. 1988;201(3):567–74. . 3418711 10.1016/0022-2836(88)90638-9

[12] Dobrov EN, Badun GA, Lukashina EV, Fedorova NV, Ksenofontov AL, Fedoseev VM, et al Tritium planigraphy comparative structural study of tobacco mosaic virus and its mutant with altered host specificity. European journal of biochemistry. 2003;270(16):3300–8. . 12899688 10.1046/j.1432-1033.2003.03680.x

[13] Baratova LA, Efimov AV, Dobrov EN, Fedorova NV, Hunt R, Badun GA, et al In situ spatial organization of Potato virus A coat protein subunits as assessed by tritium bombardment. Journal of virology. 2001;75(20):9696–702. 10.1128/JVI.75.20.9696-9702.2001 11559802 PMC114541

[14] Baratova LA, Grebenshchikov NI, Dobrov EN, Gedrovich AV, Kashirin IA, Shishkov AV, et al The organization of potato virus X coat proteins in virus particles studied by tritium planigraphy and model building. Virology. 1992;188(1):175–80. . 1566571 10.1016/0042-6822(92)90747-d

[15] Shishkov AV, Goldanskii VI, Baratova LA, Fedorova NV, Ksenofontov AL, Zhirnov OP, et al The in situ spatial arrangement of the influenza A virus matrix protein M1 assessed by tritium bombardment. Proceedings of the National Academy of Sciences of the United States of America. 1999;96(14):7827–30. 10.1073/pnas.96.14.7827 10393906 PMC22146

[16] Tsetlin VI, Alyonycheva TN, Shemyakin VV, Neiman LA, Ivanov VT. Tritium thermal activation study of bacteriorhodopsin topography. European journal of biochemistry. 1988;178(1):123–9. . 3203683 10.1111/j.1432-1033.1988.tb14437.x

[17] Agafonov DE, Kolb VA, Spirin AS. Proteins on ribosome surface: measurements of protein exposure by hot tritium bombardment technique. Proceedings of the National Academy of Sciences of the United States of America. 1997;94(24):12892–7. 10.1073/pnas.94.24.12892 9371771 PMC24234

[18] Kordyukova LV, Ksenofontov AL, Badun GA, Baratova LA, Shishkov AV. Studying liposomes by tritium bombardment. Bioscience reports. 2001;21(6):711–8. . 12166821 10.1023/a:1015572321508

[19] Shishkov AV, Ksenofontov AL, Bogacheva EN, Kordyukova LV, Badun GA, Alekseevsky AV, et al Studying the spatial organization of membrane proteins by means of tritium stratigraphy: bacteriorhodopsin in purple membrane. Bioelectrochemistry. 2002;56(1–2):147–9. . 12009462 10.1016/s1567-5394(02)00018-x

[20] Gol'danskii VI, Rumiantsev Iu M, Shishkov AV, Baratova LA, Belianova LP. [Study of the three-dimensional structure of proteins by means of tritium labeling. II. Intramolecular distribution of tritium in the N-terminal part of myoglobin and the tertiary structure of protein]. Molekuliarnaia biologiia. 1982;16(3):528–34. . 7048065

[21] Trifonova EA, Nikitin NA, Kirpichnikov MP, Karpova OV, Atabekov JG. Obtaining and characterization of spherical particles—new biogenic platforms. Moscow University Biological Sciences Bulletin. 2016;70(4):194–7. 10.3103/s0096392515040094

[22] Nikitin N, Trifonova E, Karpova O, Atabekov J. Examination of biologically active nanocomplexes by nanoparticle tracking analysis. Microscopy and microanalysis:the official journal of Microscopy Society of America, Microbeam Analysis Society, Microscopical Society of Canada. 2013;19(4):808–13. 10.1017/S1431927613000597 . 23659679

[23] Nikitin N, Trifonova E, Evtushenko E, Kirpichnikov M, Atabekov J, Karpova O. Comparative Study of Non-Enveloped Icosahedral Viruses Size. PloS one. 2015;10(11):e0142415 10.1371/journal.pone.0142415 26545232 PMC4636260

[24] Tsugita A, Scheffler JJ. A rapid method for acid hydrolysis of protein with a mixture of trifluoroacetic acid and hydrochloric acid. European journal of biochemistry. 1982;124(3):585–8. . 7106109 10.1111/j.1432-1033.1982.tb06634.x

[25] Trofimova L, Ksenofontov A, Mkrtchyan G, Graf A, Baratova L, Bunik V. Quantification of Rat Brain Amino Acids: Analysis of the Data Consistency. Current Analytical Chemistry. 2016;12(4):349–56. 10.2174/1573411011666151006220356

[26] Lukashina E, Ksenofontov A, Fedorova N, Badun G, Mukhamedzhanova A, Karpova O, et al Analysis of the role of the coat protein N-terminal segment in Potato virus X virion stability and functional activity. Molecular plant pathology. 2012;13(1):38–45. 10.1111/j.1364-3703.2011.00725.x . 21726392 PMC6638661

[27] Ksenofontov AL, Dobrov EN, Fedorova NV, Serebryakova MV, Prusov AN, Baratova LA, et al Isolated Potato Virus A coat protein possesses unusual properties and forms different short virus-like particles. Journal of biomolecular structure & dynamics. 2018;36(7):1728–38. 10.1080/07391102.2017.1333457 . 28537193

[28] Namba K, Stubbs G. Structure of tobacco mosaic virus at 3.6 A resolution: implications for assembly. Science. 1986;231(4744):1401–6. . 3952490 10.1126/science.3952490

[29] Dobrov EN, Nikitin NA, Trifonova EA, Parshina EY, Makarov VV, Maksimov GV, et al beta-structure of the coat protein subunits in spherical particles generated by tobacco mosaic virus thermal denaturation. Journal of biomolecular structure & dynamics. 2014;32(5):701–8. 10.1080/07391102.2013.788983 . 24404770

[30] McCormick AA, Palmer KE. Genetically engineered Tobacco mosaic virus as nanoparticle vaccines. Expert review of vaccines. 2008;7(1):33–41. 10.1586/14760584.7.1.33 . 18251692

[31] Fraenkel-Conrat H, Singer B. Virus reconstitution and the proof of the existence of genomic RNA. Philosophical transactions of the Royal Society of London Series B, Biological sciences. 1999;354(1383):583–6. 10.1098/rstb.1999.0409 10212937 PMC1692543

[32] Shishkov A, Bogacheva E, Fedorova N, Ksenofontov A, Badun G, Radyukhin V, et al Spatial structure peculiarities of influenza A virus matrix M1 protein in an acidic solution that simulates the internal lysosomal medium. The FEBS journal. 2011;278(24):4905–16. 10.1111/j.1742-4658.2011.08392.x . 21985378

[33] Ksenofontov AL, Paalme V, Arutyunyan AM, Semenyuk PI, Fedorova NV, Rumvolt R, et al Partially disordered structure in intravirus coat protein of potyvirus potato virus A. PloS one. 2013;8(7):e67830 10.1371/journal.pone.0067830 23844104 PMC3700898

[34] Ksenofontov AL, Parshina EY, Fedorova NV, Arutyunyan AM, Rumvolt R, Paalme V, et al Heating-induced transition of Potyvirus Potato Virus A coat protein into beta-structure. Journal of biomolecular structure & dynamics. 2016;34(2):250–8. 10.1080/07391102.2015.1022604 . 25851284

[35] Nikitin N, Ksenofontov A, Trifonova E, Arkhipenko M, Petrova E, Kondakova O, et al Thermal conversion of filamentous potato virus X into spherical particles with different properties from virions. FEBS letters. 2016;590(10):1543–51. 10.1002/1873-3468.12184 . 27098711

[36] Bruckman MA, Czapar AE, VanMeter A, Randolph LN, Steinmetz NF. Tobacco mosaic virus-based protein nanoparticles and nanorods for chemotherapy delivery targeting breast cancer. Journal of controlled release:official journal of the Controlled Release Society. 2016;231:103–13. 10.1016/j.jconrel.2016.02.045 26941034 PMC5207211

