# Supplementary figures and images for: Surface characterization of the thermal remodeling helical plant virus
Source: PLoS One. 2019 May 31;14(5):e0216905. doi: 10.1371/journal.pone.0216905 (PMC6544241; doi:10.1371/journal.pone.0216905)

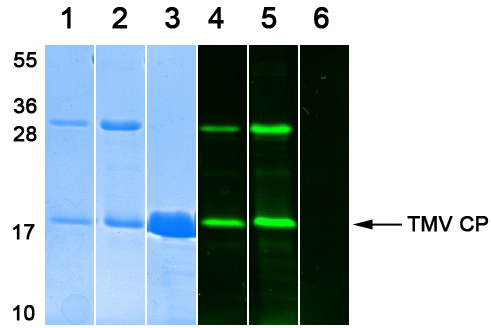

Supplement: S1 Fig — (1, 4) SPs (980 nm in diameter); (2, 5) SPs (260 nm in diameter); (3, 6) native TMV virions—control. (1–3) Coomassie Blue staining, (4–6) UV light. Positions of the molecular weight markers (kDa) are indicated at the left side. (TIF) [file pone.0216905.s001.tif]

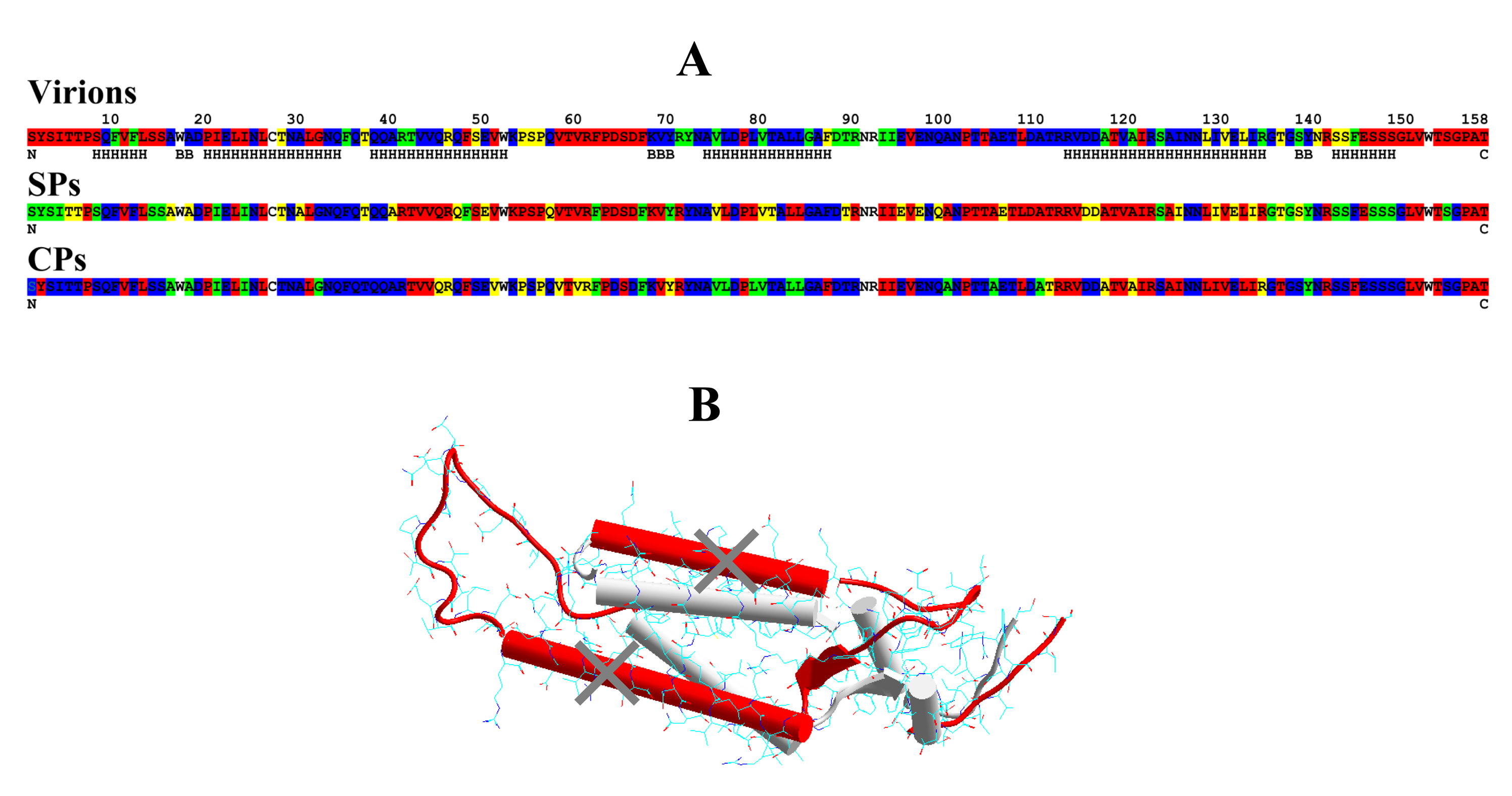

Supplement: S2 Fig — (A) The tritium incorporation into amino acid residues along the polypeptide chain of proteins SPs, TMV and isolated CP. The coloring was carried out according to the principle of rainbow and reduction of specific activity (red> yellow> green> blue–matched >0.5%, 0.5–0.4%, 0.4–0.3%, 0.3–0%, respectively. The residues lost during analysis are not colored. α-Helices (H) and β-structure (B) in U1 CP are represented. (B) The surface sites of SPs protein are indicated. The figure is based on X-ray diffraction data TMV coat protein subunit in the virion [28]. Red color shows labeled area, that possibly transformed into betta and disordered structures [29]. Region with a low level of labeling is shown in gray. (TIF) [file pone.0216905.s002.tif]
