# Supplementary material for: Surface characterization of the thermal remodeling helical plant virus
Source: PLoS One. 2019 May 31;14(5):e0216905. doi: 10.1371/journal.pone.0216905 (PMC6544241; doi:10.1371/journal.pone.0216905)
Supplement: S1 Dataset — Data set for Fig 2 and Fig 4. (PDF) [file pone.0216905.s003.pdf]

1) Data set for Fig 2 and Fig 4

The tritium label location in the TMV virion and SPs proteins upon tritium bombardment of samples.

peptides	№ res.	res.	S/nmol SPs2	S/nmol SPs3	% SPs2	% SPs3	SP avr,% SPs	vir-u1,%* Virions	% SPavr/vir
T1	1	S	578,4	1389,8	0,2	0,2	0,2	0,6	0,4
	2	Y	1024,8	773,4	0,4	0,1	0,2	1,3	0,2
	3	S	578,4	1389,8	0,2	0,2	0,2	0,6	0,4
	4	I	784,8	1192,1	0,3	0,2	0,2	0,6	0,4
	5	T	1082,8	2465,4	0,4	0,4	0,4	0,5	0,8
	6	T	1082,8	2465,4	0,4	0,4	0,4	0,5	0,8
	7	P	2072,5	3567,9	0,8	0,6	0,7	1,0	0,6
	8	S	578,4	1389,8	0,2	0,2	0,2	0,6	0,4
	9	Q	448,2	965,1	0,2	0,2	0,2	0,1	1,8
	10	F	439,2	341,1	0,2	0,1	0,1	0,3	0,4
	11	V	2415,4	8264,7	0,9	1,3	1,1	2,8	0,4
	12	F	439,2	341,1	0,2	0,1	0,1	0,3	0,4
	13	L	2180,5	6964,4	0,8	1,1	0,9	0,5	1,9
	14	S	578,5	1389,8	0,2	0,2	0,2	0,6	0,4
	15	S	578,5	1389,8	0,2	0,2	0,2	0,6	0,4
	16	A	664,5	2669,6	0,2	0,4	0,3	0,2	2,2
	17	W	0,0	0,0	0,0	0,0	0,0		
	18	A	664,5	2669,6	0,2	0,4	0,3	0,2	2,2
	19	D	197,3	617,2	0,1	0,1	0,1	0,1	1,2
	20	P	2072,0	3567,9	0,8	0,6	0,7	1,0	0,6
	21	I	784,8	1192,1	0,3	0,2	0,2	0,6	0,4
	22	E	129,6	420,0	0,0	0,1	0,1	0,1	0,6
	23	L	2180,5	6964,4	0,8	1,1	0,9	0,5	1,9
	24	I	784,8	1192,1	0,3	0,2	0,2	0,6	0,4
	25	N	196,8	617,2	0,1	0,1	0,1	0,1	1,2
	26	L	2180,5	6964,4	0,8	1,1	0,9	0,5	1,9
	27	C	0,0	0,0	0,0	0,0	0,0		
	28	T	1082,4	2465,4	0,4	0,4	0,4	0,5	0,8
	29	N	197,3	617,2	0,1	0,1	0,1	0,1	1,2
	30	A	664,8	2669,6	0,2	0,4	0,3	0,2	2,2
	31	L	2180,5	6964,4	0,8	1,1	0,9	0,5	1,9
	32	G	129,6	420,0	0,0	0,1	0,1	1,0	0,1
	33	N	197,3	617,2	0,1	0,1	0,1	0,1	1,2
	34	Q	448,2	965,1	0,2	0,2	0,2	0,1	1,8
	35	F	439,2	341,1	0,2	0,1	0,1	0,3	0,4
	36	Q	448,2	965,1	0,2	0,2	0,2	0,1	1,8
	37	T	1082,4	2465,4	0,4	0,4	0,4	0,5	0,8
	38	Q	448,2	965,1	0,2	0,2	0,2	0,1	1,8
	39	Q	448,2	965,1	0,2	0,2	0,2	0,1	1,8
	40	A	664,8	2669,6	0,2	0,4	0,3	0,2	2,2
	41	R	2659,2	5236,8	1,0	0,8	0,9	0,2	4,5
T2	42	T	1035,6	4905,4	0,4	0,8	0,6	0,3	2,3
	43	V	2548,9	11811,4	0,9	1,9	1,4	0,1	12,7
	44	V	2548,9	11811,4	0,9	1,9	1,4	0,1	12,7

	**45**	**Q**	854,4	6457,1	0,3	1,0	0,7	0,2	4,5
	**46**	**R**	1882,9	3861,0	0,7	0,6	0,6	0,3	2,2
**T3**	**47**	**Q**	1199,9	1648,4	0,4	0,3	0,3	0,1	5,8
	**48**	**F**	828,5	1513,2	0,3	0,2	0,3	0,9	0,3
	**49**	**S**	2636,0	3870,7	1,0	0,6	0,8	0,4	1,9
	**50**	**Q**	1199,9	1648,4	0,4	0,3	0,3	0,1	5,8
	**51**	**V**	2614,1	5392,6	0,9	0,9	0,9	0,7	1,2
	**52**	**W**	0,0	0,0	0,0	0,0	0,0		
	**53**	**K**	1621,1	2768,2	0,6	0,4	0,5	0,0	12,9
	**54**	**P**	4617,5	6881,9	1,7	1,1	1,4	0,4	3,1
	**55**	**S**	2636,0	3870,7	1,0	0,6	0,8	0,4	1,9
	**56**	**P**	4617,5	6881,9	1,7	1,1	1,4	0,4	3,1
	**57**	**Q**	1200,0	1648,4	0,4	0,3	0,3	0,1	5,8
	**58**	**V**	2614,1	5392,6	0,9	0,9	0,9	0,7	1,2
	**59**	**T**	4880,3	8226,9	1,8	1,3	1,5	3,9	0,4
	**60**	**V**	2614,1	5392,6	0,9	0,9	0,9	0,7	1,2
	**61**	**R**	1828,2	2582,3	0,7	0,4	0,5	1,3	0,4
**T4**	**62**	**F**	610,8	1789,8	0,2	0,3	0,3	0,6	0,4
	**63**	**P**	7127,5	23035,6	2,6	3,7	3,1	2,2	1,5
	**64**	**D**	776,1	5041,5	0,3	0,8	0,5	0,6	0,9
	**65**	**S**	2013,8	7925,6	0,7	1,3	1,0	4,1	0,2
	**66**	**D**	776,4	5041,5	0,3	0,8	0,5	0,6	0,9
	**67**	**F**	611,4	1789,8	0,2	0,3	0,3	0,6	0,4
	**68**	**K**	1281,9	4028,7	0,5	0,6	0,6	0,1	6,1
**T5**	**69**	**V**	2757,7	7118,9	1,0	1,1	1,1	0,2	5,6
	**70**	**Y**	965,7	1020,0	0,4	0,2	0,3	0,1	3,7
	**71**	**R**	6019,8	4130,1	2,2	0,7	1,4	0,2	6,2
**T6**	**72**	**Y**	505,6	234,2	0,2	0,0	0,1	0,2	0,5
	**73**	**N**	212,1	635,0	0,1	0,1	0,1	0,0	2,2
	**74**	**A**	595,7	819,2	0,2	0,1	0,2	0,1	1,9
	**75**	**V**	1182,1	1619,0	0,4	0,3	0,3	0,3	1,4
	**76**	**L**	1714,0	2782,1	0,6	0,4	0,5	0,1	4,1
	**77**	**D**	212,4	635,0	0,1	0,1	0,1	0,0	2,2
	**78**	**P**	2409,3	4320,1	0,9	0,7	0,8	0,9	0,9
	**79**	**L**	1714,0	2782,1	0,6	0,4	0,5	0,1	4,1
	**80**	**V**	1182,1	1619,0	0,4	0,3	0,3	0,3	1,4
	**81**	**T**	1390,8	1863,9	0,5	0,3	0,4	0,2	2,1
	**82**	**A**	595,7	819,2	0,2	0,1	0,2	0,1	1,9
	**83**	**L**	1714,0	2782,1	0,6	0,4	0,5	0,1	4,1
	**84**	**L**	1714,0	2782,1	0,6	0,4	0,5	0,1	4,1
	**85**	**G**	201,5	140,7	0,1	0,0	0,0	0,2	0,2
	**86**	**A**	595,7	819,2	0,2	0,1	0,2	0,1	1,9
	**87**	**F**	631,9	875,2	0,2	0,1	0,2	0,4	0,4
	**88**	**D**	212,1	635,0	0,1	0,1	0,1	0,0	2,2
	**89**	**T**	1390,8	1863,9	0,5	0,3	0,4	0,2	2,1
	**90**	**R**	2261,6	3973,7	0,8	0,6	0,7	0,3	2,7
**T7**	**91**	**N**	0,0	0,0	0,0	0,0	0,0		
	**92**	**R**	0,0	0,0	0,0	0,0	0,0		
**T8**	**93**	**I**	2084,2	6301,3	0,8	1,0	0,9		
	**94**	**I**	2084,2	6301,3	0,8	1,0	0,9	0,2	4,4

	**95**	**E**	1559,9	1950,6	0,6	0,3	0,4	0,0	11,0
	**96**	**V**	5440,5	12306,6	2,0	2,0	2,0	0,8	2,6
	**97**	**E**	1559,9	1950,6	0,6	0,3	0,4	0,0	11,0
	**98**	**N**	521,4	987,9	0,2	0,2	0,2	0,1	2,5
	**99**	**Q**	1559,9	1950,6	0,6	0,3	0,4	0,0	11,0
	**100**	**A**	2317,7	4572,1	0,8	0,7	0,8	0,1	11,2
	**101**	**N**	521,4	987,9	0,2	0,2	0,2	0,1	2,5
	**102**	**P**	11409,5	23395,0	4,1	3,7	3,9	1,1	3,7
	**103**	**T**	3143,1	8250,9	1,1	1,3	1,2	0,2	6,8
	**104**	**T**	3143,1	8250,9	1,1	1,3	1,2	0,2	6,8
	**105**	**A**	2317,7	4572,1	0,8	0,7	0,8	0,1	11,2
	**106**	**E**	1559,9	1950,6	0,6	0,3	0,4	0,0	11,0
	**107**	**T**	3143,1	8250,9	1,1	1,3	1,2	0,2	6,8
	**108**	**L**	11602,7	36307,4	4,2	5,8	5,0	0,8	6,4
	**109**	**D**	2317,7	987,9	0,8	0,2	0,5	0,1	7,1
	**110**	**A**	2317,3	4572,1	0,8	0,7	0,8	0,1	11,2
	**111**	**T**	3143,1	8250,9	1,1	1,3	1,2	0,2	6,8
	**112**	**R**	2343,0	4292,2	0,9	0,7	0,8	0,2	4,8
**T9**	**113**	**R**	1969,6	7514,2	0,7	1,2	1,0	0,1	6,8
	**114**	**V**	5324,6	12855,0	1,9	2,0	2,0	0,2	13,3
	**115**	**D**	1039,1	3089,8	0,4	0,5	0,4	0,1	6,2
	**116**	**D**	1039,1	3089,8	0,4	0,5	0,4	0,1	6,2
	**117**	**A**	2406,1	5021,3	0,9	0,8	0,8	0,3	3,0
	**118**	**T**	9546,8	18305,0	3,5	2,9	3,2	0,5	6,4
	**119**	**V**	5324,6	12855,0	1,9	2,0	2,0	0,2	13,3
	**120**	**A**	2406,1	5021,3	0,9	0,8	0,8	0,3	3,0
	**121**	**I**	3934,2	9056,4	1,4	1,4	1,4	0,6	2,3
	**122**	**R**	1969,6	7514,2	0,7	1,2	1,0	0,1	6,8
**T10**	**123**	**S**	1005,6	1185,1	0,4	0,2	0,3	0,3	1,0
	**124**	**A**	1420,9	3575,6	0,5	0,6	0,5	0,1	4,2
	**125**	**I**	967,2	3393,0	0,4	0,5	0,4	0,1	3,2
	**126**	**N**	296,4	566,9	0,1	0,1	0,1	0,0	2,5
	**127**	**N**	296,4	566,9	0,1	0,1	0,1	0,0	2,5
	**128**	**L**	3242,5	10124,2	1,2	1,6	1,4	0,4	4,0
	**129**	**I**	967,2	3393,0	0,4	0,5	0,4	0,1	3,2
	**130**	**V**	1682,5	3781,2	0,6	0,6	0,6	0,4	1,6
	**131**	**E**	913,2	2791,2	0,3	0,4	0,4	0,1	5,5
	**132**	**L**	3242,5	10124,2	1,2	1,6	1,4	0,4	4,0
	**133**	**I**	967,2	3393,0	0,4	0,5	0,4	0,1	3,2
	**134**	**R**	1960,9	4100,0	0,7	0,7	0,7	0,2	3,0
**T11**	**135**	**G**	532,7	1260,8	0,2	0,2	0,2	0,1	1,8
	**136**	**T**	798,4	3825,4	0,3	0,6	0,4	0,4	1,3
	**137**	**G**	532,7	1260,8	0,2	0,2	0,2	0,1	1,8
	**138**	**S**	1335,8	3108,3	0,5	0,5	0,5	0,2	2,4
	**139**	**Y**	780,5	1163,1	0,3	0,2	0,2	0,2	1,4
	**140**	**N**	207,7	947,4	0,1	0,2	0,1	0,3	0,4
	**141**	**R**	1449,6	4687,9	0,5	0,7	0,6	3,3	0,2
**T12**	**142**	**S**	661,1	1217,9	0,2	0,2	0,2	0,9	0,3
	**143**	**S**	661,1	1217,9	0,2	0,2	0,2	0,9	0,3
	**144**	**F**	455,4	908,7	0,2	0,1	0,2	0,2	0,7

	145	E	2476,6	4179,9	0,9	0,7	0,8	3,0	0,3
	146	S	661,2	1217,9	0,2	0,2	0,2	0,9	0,3
	147	S	661,2	1217,9	0,2	0,2	0,2	0,9	0,3
	148	S	661,2	1217,9	0,2	0,2	0,2	0,9	0,3
	149	G	581,7	452,3	0,2	0,1	0,1	1,5	0,1
	150	L	3630,5	11264,8	1,3	1,8	1,6	1,5	1,0
	151	V	4649,2	8989,3	1,7	1,4	1,6	3,7	0,4
	152	W	0,0	0,0	0,0	0,0	0,0		
	153	T	2378,2	2045,9	0,9	0,3	0,6	3,2	0,2
	154	S	661,2	1217,9	0,2	0,2	0,2	0,9	0,3
	155	G	582,0	452,3	0,2	0,1	0,1	1,5	0,1
	156	P	7052,6	14850,4	2,6	2,4	2,5	19,7	0,1
	157	A	2392,8	3121,4	0,9	0,5	0,7	1,7	0,4
	158	T	2378,5	2045,9	0,9	0,3	0,6	3,2	0,2
		Sum	275356,9	628557,5	100	100	100	100,08	

2) Data set for Fig 4.

Total radioactivity of amino acid residues of protein in the SPs and virions of TMV.

Amino acids	res.	№ res.	SPs	virion*	SP/vir
Hydrophobic/aliphatic	I	9	5,24	3,05	1,7
	L	12	15,27	5,49	2,8
	V	14	16,48	11,02	1,5
	A	14	7,10	3,49	2,0
aromatic	F	8	1,44	3,49	0,4
	Y	4	0,85	1,74	0,5
Hydrophophilic	S	16	5,72	13,53	0,4
	T	15	14,23	14,37	1,0
	N	10	1,00	0,79	1,3
	Q	9	2,95	0,82	3,6
Basic/Acid	E	7	2,49	3,19	0,8
	D	8	2,72	1,52	1,8
	K	2	1,07	0,13	8,2
	R	11	8,23	6,30	1,3
Small	G	6	0,84	4,46	0,2
	P	8	14,39	26,69	0,5

* Data for the TMV virus are those published in our previous work [1]

Dobrov EN, Badun GA, Lukashina EV, Fedorova NV, Ksenofontov AL, Fedoseev VM, et al. Tritium planigraphy comparative structural study of tobacco mosaic virus and its mutant with altered host specificity. European journal of biochemistry. 2003;270(16):3300-8.
